# Large-Scale Investigation of Soybean Gene Functions by Overexpressing a Full-Length Soybean cDNA Library in *Arabidopsis*

**DOI:** 10.3389/fpls.2018.00631

**Published:** 2018-05-09

**Authors:** Xiang Li, Lei Huang, Jianhua Lu, Yihui Cheng, Qingbo You, Lijun Wang, Xuejiao Song, Xinan Zhou, Yongqing Jiao

**Affiliations:** ^1^Key Laboratory of Biology and Genetic Improvement of Oil Crops, Ministry of Agriculture, Oil Crops Research Institute, Chinese Academy of Agricultural Sciences, Wuhan, China; ^2^The College of Life Science, Yangtze University, Jingzhou, China; ^3^State Key Laboratory of Crop Biology, College of Agronomy, Shandong Agricultural University, Tai’an, China; ^4^Collaborative Innovation Center of Henan Grain Crops, College of Agronomy, Henan Agricultural University, Zhengzhou, China

**Keywords:** cDNA library, functional genomics, large-scale, gene mining, soybean

## Abstract

Molecular breeding has become an important approach for crop improvement, and a prerequisite for molecular breeding is elucidation of the functions of genetic loci or genes. Soybean is one of the most important food and oil crops worldwide. However, due to the difficulty of genetic transformation in soybean, studies of its functional genomics lag far behind those of other crops such as rice, which severely impairs the progress of molecular improvement in soybean. Here, we describe an effective large-scale strategy to investigate the functions of soybean genes via overexpression of a full-length soybean cDNA library in *Arabidopsis*. The overexpression vector *pJL12* was modified for use in the construction of a normalized full-length cDNA library. The constructed cDNA library showed good quality; repetitive clones represented approximately 4%, insertion fragments were approximately 2.2 kb, and the full-length rate was approximately 98%. This cDNA library was then overexpressed in *Arabidopsis*, and approximately 2000 transgenic lines were preliminarily obtained. Phenotypic analyses of the positive T_1_ transgenic plants showed that more than 5% of the T_1_ transgenic lines displayed abnormal developmental phenotypes, and approximately 1% of the transgenic lines exhibited potentially favorable traits. We randomly amplified 4 genes with obvious phenotypes (enlarged seeds, yellowish leaves, more branches, and dense siliques) and repeated the transgenic analyses in *Arabidopsis*. Subsequent phenotypic observation demonstrated that these phenotypes were indeed due to the overexpression of soybean genes. We believe our strategy represents an effective large-scale approach to investigate the functions of soybean genes and further reveal genes favorable for molecular improvement in soybean.

## Introduction

Conventional crop breeding is challenging and often takes a great deal of time. Molecular breeding approaches, such as marker-assisted or transgenic breeding, have become an important approach for current crop improvement. The prerequisite for molecular breeding is elucidation of the functions of genetic loci or genes. Among the different crop species where molecular breeding has been successfully applied, rice is a good example. For seed-related traits, several loci affecting grain size have been successfully identified and elucidated, such as *GW7* (Wang S. et al., 2015), *GW2* (Song et al., 2007) and *GL7* (Si et al., 2016). Further transgenic application of these genes greatly enhanced rice grain yield (Song et al., 2007; Wang Y. et al., 2015; Si et al., 2016). Plant architecture is another important factor affecting grain yield. *Ideal Plant Architecture 1* (*IPA1*) is an important dominant gene that reduces tiller number and enlarges panicles (Jiao et al., 2010). Practical application of this gene could greatly enhance grain yield compared to that of the control.

Soybean (*Glycine max* (L.) Merr.) is one of the most important food and oil crops worldwide, providing abundant proteins, oil and nutrition sources for animal feed and the human diet (Lam et al., 2010; Korir et al., 2013). At present, over one-third of edible oils and two-thirds of protein meal in the world are derived from soybean (Sobhanian et al., 2010). Soybean researchers have made some progress in soybean functional genomics through either forward genetic or reverse genetic studies. For example, a series of soybean gene functions have been elucidated, including *GmphyA1* and *GmphyA2* (Liu et al., 2008), *Arabidopsis thaliana EARLY FLOWERING 3* (*ELF3*) homologous *J* locus (Lu et al., 2017), *GmGIP1* (Ma et al., 2017), *GmAP1* (Chi et al., 2011), and *GmZF351* (Li et al., 2017). However, due to the very large and complex genome, low transformation efficiency, and long transgenic process in soybean, studies of soybean functional genomics have lagged far behind those in other crops, such as rice (*Oryza sativa*). In addition, soybean is an ancient tetraploid plant with many redundant genes, which seriously influence the elucidation of gene function by means of studying loss-of-function soybean mutants. To date, many kinds of RNA and protein profiling in soybean have been conducted, and numerous regulated genes have been identified (Smith-Hammond et al., 2014; Lanubile et al., 2015; Yu et al., 2016). However, confirmation of the functions of these genes has been rare, let alone the application of these genes in molecular improvement. Therefore, a strategy is needed to conduct large-scale and efficient studies in soybean functional genomics and, especially, to mine a large number of candidate favorable genes for soybean genetic improvement.

*Arabidopsis* is a model plant for studies of dicotyledons and thus represents an important intermediate tool to confirm the functions of soybean genes (Liu et al., 2014; Wang F. et al., 2015; Wei et al., 2015; Pan et al., 2017). In this study, we overexpressed a full-length soybean cDNA library in *Arabidopsis* and investigated the functions of soybean genes by evaluating the phenotypes of transgenic *Arabidopsis*. Our strategy could provide an efficient and high-throughput approach to mining candidate favorable soybean genes that could facilitate soybean molecular improvement.

## Materials and Methods

### Plant Materials and Growth Conditions

The roots, stems, leaves and shoot apices of soybean cultivar Williams 82 were used as materials for RNA extraction. *Arabidopsis* ecotype Col-0 was used as transformation recipient. *Arabidopsis* Col-0 was planted in a greenhouse with a temperature of 23°C, humidity of approximately 75%, and a 16 h light and 8 h dark photoperiod.

### Vector Modification and Construction of the Full-Length cDNA Library

For vector modification, we first analyzed the sequence of vector *pJL12* and found an *Sfi*I recognition site (downstream of the NOS terminator at position 3859). However, there was no *Sfi*I site downstream of the 35S promoter. Thus, we needed to mutate the existing *Sfi*I site (at position 3859) and add another downstream of the 35S promoter for the integration of cDNA inserts. Briefly, we artificially synthesized a 1397 nucleotide fragment from position 2563 to 3907 in *pJL12*, which contained *BamH*I and *Sse232*I adapters. This fragment was designed to mutate the *Sfi*I recognition site at position 3896 by changing G to T and introduce a new *Sfi*I site downstream of the 35S promoter, where the sequence ACTAGTTCTAGA was replaced by GGCCATTACGGCCAAGCTTGATATCGGCCGCCTCGGCC. Next, the *pJL12* fragment from position 2563 (*BamHI*) to 3927 (*Sse232I*) was replaced by the newly synthesized fragment using *BamH*I and *Sse232*I digestion and T4 DNA ligase linkage.

For cDNA synthesis, RNA was extracted using TRIzol reagent (Invitrogen) and reverse-transcribed by a PrimeScript reagent kit (cat#RR047A, TaKaRa). Reverse transcription primers were a combination of 5SM4Z, 5SM4ZA, and 5SM4ZB in a ratio of 1:1:1 (5SM4Z: GCC ATT ACG GCC AAG TTA C XXXX X-3′, 5SM4ZA: GCC ATT ACG GCC AAG TTA C XXXX-3′, 5SM4ZB: GCC ATT ACG GCC AAG TTA C XXX-3′, X = modified G). Subsequently, normalization was performed according to Patanjali et al. (1991), Soares et al. (1994), Zhang et al. (2005), and Li et al. (2012). Later, the normalized cDNAs were integrated into the *pJL12* vector using T4 DNA ligase (Thermo Scientific) and transformed into DH5α for propagation and storage.

### Large-Scale Genetic Transformation of the cDNA Library Into *Arabidopsis*

The concentrations of the plasmids harboring the cDNA library were measured by a spectrophotometer (Nanodrop, Thermo fisher) and diluted to ensure values between 100 and 200 ng/μl. Then, 1 μl of each plasmid was transformed into *Agrobacterium* strain GV3101 by an electrotransformation apparatus (MicroPulser, Bio-Rad). After *Agrobacterium* colonies appeared, all colonies were scraped into 1 L LB medium (kanamycin 30 ng/μl, rifampicin 50 ng/μl) and propagated in an incubator. To avoid the possibility of growth priority in a prolonged culture period, the culture time for *Agrobacterium* propagation was limited to 4∼6 h. The transformation experiment was performed as described by Clough and Bent (1998), except that we used 500 μl Silwet L-77 per liter LB medium when performing the transformation, because Silwet L-77 could greatly improve the transformation rate (Martinez-Trujillo et al., 2004).

### Phenotypic Evaluation and Genetic Analyses of Transgenic *Arabidopsis*

The transformed seeds overexpressing the full-length cDNA library were planted in a greenhouse. After screening with Basta, the surviving positive seedlings were compared to the seedlings expressing the empty vector at all developmental stages.

For the screening of homozygous transgenic lines, approximately 30 seeds derived from T_2_ plants were plated on Basta selective (1:1000 ratio dilution) plates, and the seeds of the plants whose seeds showed a 3:1 survival ratio were harvested. The phenotypes of the homozygous transgenic lines were further investigated without Basta selection.

For the A13, B12, C15 and D70 overexpression plasmids, the primers A13-f/A13-r, B12-f/B12-r, C15-f/C15-r and D70-f/D70-r were used to amplify each gene individually (Supplementary File 1). The PCR products were integrated into linearized *pJL12* vector using the ClonExpress II One Step Cloning Kit (Vazyme, cat#c112) after digestion by the restriction enzyme *BamH*I. The correct constructs were retransformed into Col-0 to validate the observed phenotypes.

### Chlorophyll Content Analysis

The total chlorophyll of the 4th and 5th rosette leaves was extracted as described in a previous report (Sakuraba et al., 2014b). Subsequently, the chlorophyll content was quantified according to another published report (Lichtenthaler, 1987).

## Results

### Construction of Soybean Full-Length cDNA Library

**FIGURE 1:**
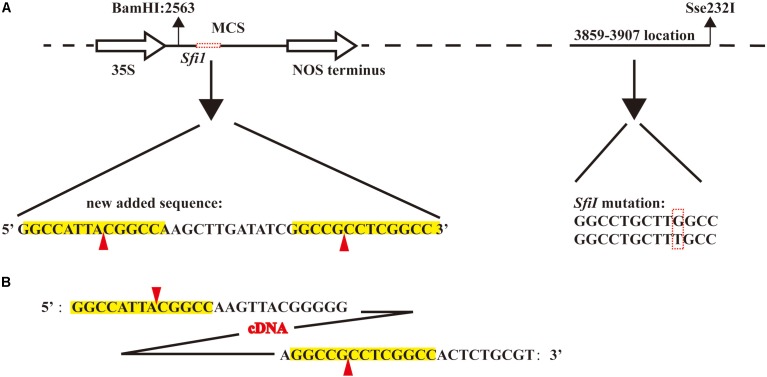
Construction of soybean full-length cDNA overexpression library. **(A)** Schematic of modified *pJL12* vector. The solid and empty arrows represent the 35S promoter and NOS terminator, respectively. The black dashed line represents the *pJL12* backbone sequence. The horizontal red dashed box indicates the artificially added sequence, which contains two *Sfi*I recognition sequences, and the vertical red dashed box indicates the mutated *Sfi*I base sequence. The red arrow indicates the *Sfi*I cleavage site, and the yellow background represents the *Sfi*I recognition site. **(B)** Reverse-transcribed and normalized cDNA sequence. The yellow background represents the *Sfi*I recognition site, and the red arrow indicates the *Sfi*I cleavage site.

The vector *pJL12*, harboring the 35S promoter, is widely used in studies of functional genomics (Qiu et al., 2002, 2008a,b; Collins et al., 2003; Zou et al., 2011; Guo et al., 2014). To make this vector suitable for the construction of a full-length cDNA library, we first modified this vector. Because the cohesive ends produced by *Sfi*I digestion were required, we first analyzed the sequence of the original *pJL12* vector and found an *Sfi*I restriction site in *pJL12* (position 3859–3907, **Figure 1A**). However, downstream of the 35S promoter in the MCS position, there was no *Sfi*I site. Therefore, we needed to mutate the existing *Sfi*I restriction site (G to T transition) and add another in the MCS (**Figure 1A**). Considering the above, a nucleotide fragment with a length of 1397 containing the *BamH*I and *Sse232*I recognition sites was artificially synthesized (Supplementary File 2), in which one *Sfi*I was mutated by transitioning G to T at position 3859–3907 and another *Sfi*I was introduced downstream of the 35S promoter at position 2568. This fragment was later integrated into *pJL12* by *BamH*I and *Sse232*I digestion. Thus, the modified *pJL12* vector could allow the insertion of the cDNA library after digestion by the *Sfi*I restriction enzyme and the overexpression of the library in plants through the 35S promoter.

We collected the roots, stems, leaves and shoot apices of Williams 82 plants for RNA extraction. Different adapters with *Sfi*I cohesive ends at the 5′ and 3′ ends were used to perform reverse transcription (see section “Materials and methods”). Next, the cDNA was digested with *Sfi*I and then separated by use of agarose gel electrophoresis. The approximately 2 kb fragments were recovered, normalized and ligated to the modified *pJL12* vector (**Figure 1B**). In the end, we transformed the cDNA library into DH5α. In total, we obtained approximately 10^6^ clones and retained all of the clones for propagation and storage (**Table 1**).

**Table 1:** Assessment summary of the constructed soybean full-length cDNA overexpression library.

	Total clones	Non-insert clone rate	Average insert size
cDNA library	1 × 10^6^	0%	2.2 kb

### Assessment of cDNA Library Quality

**FIGURE 2:**
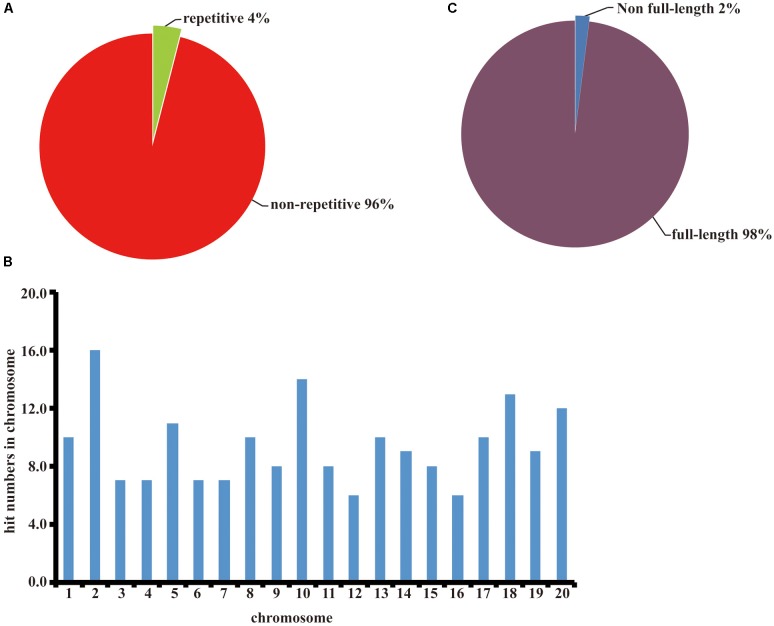
Quality assessment of the constructed soybean full-length cDNA overexpression library. **(A)** Repetitive frequency analysis of the constructed cDNA library. **(B)** Full-length proportion analysis of the constructed cDNA library. **(C)** Genome coverage analysis of the constructed cDNA library.

We randomly picked 96 colonies and used primers pJL12-f (in the 35S promoter) and pJL12-r (in the NOS terminator) to amplify the cDNA inserts. All 96 colonies were successfully amplified, which showed that each clone carried a cDNA insertion (Supplementary File 3). To evaluate the normalization rate of the constructed cDNA library, we sequenced the 96 PCR products. As shown in **Figure 2A**, non-repetitive cDNA sequences in the constructed cDNA library were approximately 96% and repetitive sequences were approximately 4%. To evaluate the genome coverage, we blasted the sequenced results against the Wm82.a2.v1 Transcript Sequences database^1^. Because soybean is a tetraploid plant, most of our sequencing data could match more than one gene locus. We retrieved gene matches with more than 95% identities for further analyses. All of the chromosomes had similar cDNA hit numbers (**Figure 2B**), which suggested that our constructed soybean full-length overexpressed cDNA library had good coverage. Later, we evaluated the full-length rate of our constructed cDNA library. Among the sequenced data, approximately 98% included full-length sequences, indicating that the majority of the inserts in the cDNA library were intact (**Figure 2C**). We also estimated the average insert size of our constructed cDNA library, which was approximately 2.2 kb (**Table 1**). Based on the above results, we concluded that the constructed full-length soybean cDNA library had high quality with high genome coverage, low repetitive frequency and high full-length ratio, and it could be used for further study.

### Transformation and Evaluation of Transgenic *Arabidopsis*

**FIGURE 3:**
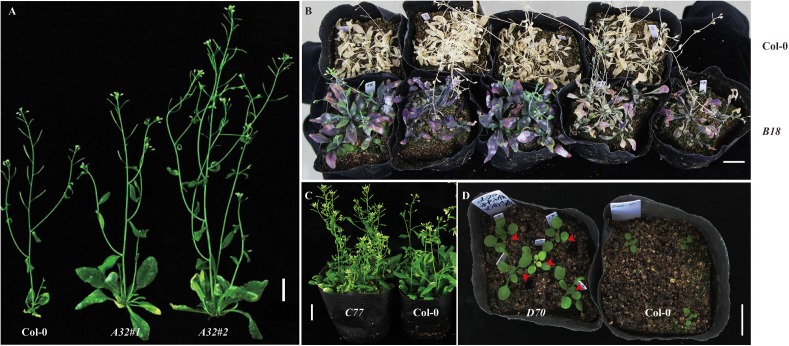
Homozygous T_2_ transformed *Arabidopsis* showed potential favorable traits. **(A)** *A32* promotes plant growth. Two homozygous *A32* lines showed more vigorous growth than that of Col-0. **(B)** *B18* enhances drought resistance. Five homozygous *B18* transgenic lines showed stronger drought resistance than that of Col-0. **(C)** *C77* increases fruit density. Several homozygous *C77* transgenic lines exhibited higher silique density than Col-0. **(D)** *D70* exhibits enlarged seed size. Five homozygous D70 transgenic lines showed larger cotyledons than those of Col-0. The red arrows indicate enlarged cotyledons. Bar = 2 cm.

We extracted the plasmids from the cDNA library and transformed them into *Agrobacterium* cells. Next, the library was transformed into *Arabidopsis* through *Agrobacterium tumefaciens*–mediated transformation method with a slight modification (see section “Materials and methods”) (Clough and Bent, 1998). To obtain T_1_ positive transgenic lines overexpressing the soybean full-length cDNA library, T_0_ generation seeds were harvested and sown in a greenhouse, and Basta (1:1000) was sprayed for selection. In total, we obtained nearly 2000 T_1_ positive transgenic lines (**Table 2**). To observe phenotypes in the T_1_ transgenic lines, empty vector *pJL*12 (modified)–transformed lines were sown in parallel with positive cDNA-transformed T_1_ seedlings. Many of the T_1_ positive seedlings exhibited abnormal phenotypes (approximately 5%), including late flowering, yellowish color, loss of SAM (shoot apical meristem) activity, early senescence, more primary and secondary branches, curved leaves, smooth stems and early flowering (Supplementary File 4 and **Table 2**), whereas another 1% exhibited favorable traits, including large seeds, high silique density, strong drought resistance and vigorous growth appearance (**Figure 3** and **Table 2**).

**Table 2:** Summary of phenotypes of T_1_ positive *Arabidopsis* lines transformed with the soybean full-length cDNA overexpression library.

Phenotypes	Lines found
Early flowering	16
Yellowish color	3
Early senescence	8
Thin and early flowering	18
Loss of SAM activity	12
Late flowering	1
More branches	9
Curved leaf	7
Smooth stem and no cauline leaf	1
Infertile	9
Yellow seed coat	2
Short height	1
Lodged stem	2
Slow germination	2
Large seed size	8
Vigorous growth	3
Stronger stem	3
High silique density	1
Drought resistant	3
Total obtained lines	∼2000
Negative trait lines	92
Favorable trait lines	18

To inspect the distribution of cDNA inserts in the soybean genome, we extracted DNA from 96 randomly selected individual T_1_ seedlings and amplified their cDNA inserts using the primers pJL12-f and pJL12-r. Most of the 96 transgenic plants had successfully amplified products (Supplementary File 5A). Then, the amplified products were sequenced and blasted against the Wm82.a2.v1 Transcript Sequences database. The sequencing data showed that 97% of the cDNA inserts were non-repetitive, and all of the sequenced data were full-length (Supplementary File 5B). To examine the coverage of the cDNA inserts, we analyzed the distribution of sequencing data in the soybean genome. The results showed that all 20 soybean chromosomes had good match numbers (Supplementary File 5C).

### Validation of Phenotypes of T_1_ Transgenic *Arabidopsis* by Retransformation of Corresponding Genes

To confirm whether the phenotypes of transgenic *Arabidopsis* were caused by the ectopic expression of soybean genes, we randomly selected 4 lines with obvious phenotypes for further study.

**FIGURE 4:**
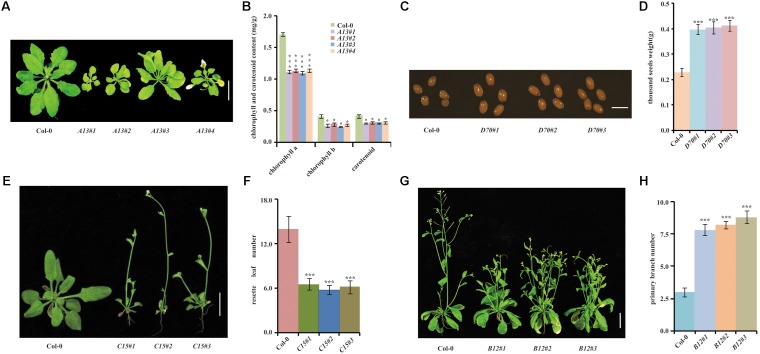
Phenotypic validation of 4 randomly selected transgenic lines by genetic retransformation. **(A)** Leaf phenotype of retransformed *A13* homozygous lines and Col-0 control. **(B)** Chlorophyll and carotenoid contents of retransformed *A13* homozygous lines and Col-0. **(C)** Photograph of seed size in retransformed *D70* homozygous lines and Col-0 control. **(D)** Thousand seed weights of retransformed *D70* homozygous lines and Col-0. **(E)** Phenotypes of retransformed *C15* homozygous lines and Col-0 plants. **(F)** Rosette leaf numbers in retransformed *C15* homozygous lines and Col-0 at bolting. **(G)** Branching phenotypes of retransformed *B12* homozygous lines and Col-0 plants. **(H)** Primary branch statistical analysis in Col-0 and retransformed *B12* homozygous lines. Bar = 2 cm, *n* ≥ 10. (^∗∗∗^*p* < 0.01, ^∗^*p* < 0.05, Student’s *t*-test).

A13 homozygous transgenic plants displayed a yellowish phenotype (**Figures 4A,B**). The sequence analysis of the cDNA fragments showed that the A13 yellowish phenotype was caused by *Glyma.06g119500*, a homolog of *Arabidopsis SGR1* (Shimoda et al., 2016). We reconstructed the overexpression vector for *Glyma.06g119500* and transformed it into *Arabidopsis*. Phenotypic analysis showed that all homozygous transgenic lines exhibited a yellowing leaf phenotype (**Figures 4A,B**) consistent with the one from the transgenic line overexpressing the cDNA library.

B12 homozygous transgenic plants displayed a dwarfing and highly branched phenotype (**Figures 4C,D**). Sequence analysis showed that it might be caused by a predicted *ANDROGEN INDUCED INHIBITOR OF PROLIFERATION* (*AS3*) / *PDS5*-*RELATED* gene, *Glyma.06g062700*. To validate whether the B12 phenotype was caused by overexpression of *Glyma.06g062700*, we retransformed *Glyma.06g062700* into *Arabidopsis*. Among 26 homozygous lines, 19 displayed obvious dwarfing and highly branched phenotypes (**Figures 4C,D**), suggesting that the B12 phenotype was due to overexpression of *Glyma.06g062700*.

C15 T_1_ transgenic plants were thin, with small stature and early flowering (**Figures 4E,F**). Sequence analysis revealed that the inserted gene was *Glyma.11g199100*, a nucleic acid binding protein with ATP-dependent RNA helicase activity. Retransformed *Arabidopsis* lines with overexpression of *Glyma.11g199100* also showed the same thin, small and early-flowering phenotype (**Figures 4E,F**), demonstrating that the C15 phenotype was caused by overexpression of *Glyma.11g199100*.

The D70 T_1_ transgenic line displayed larger seeds (**Figures 4G,H**). Sequence analysis showed that the D70 insertion gene was *Glyma.05g231900*, which encoded an aldehyde dehydrogenase. To validate whether the large seeds in D70 were caused by *Glyma.05g231900*, we retransformed the overexpression vectors of *Glyma.05g231900* into *Arabidopsis*. The results showed that 19 out of 24 homozygous lines showed larger seed sizes than those of the controls (**Figures 4G,H**), demonstrating that the aldehyde dehydrogenase *Glyma.05g231900* exerts a specific influence on seed size.

Taking the above results together, we concluded that the phenotypes found in the T_1_ generation plants were most likely caused by the overexpression of the genes from the soybean full-length cDNA overexpression library.

## Discussion

Soybean is an important crop that provides a well-balanced source of protein and oil. In addition, most of the components of soybean, such as α-linolenic acid and isoflavones, have beneficial health effects (Xia et al., 2013). However, studies of the functional genomics of soybean have lagged far behind those of other crops because of the relatively low efficiency and long transformation period and the relative complexity of the genome (Schmutz et al., 2010). Although some genes have been cloned (Lee et al., 2004; Gao et al., 2005; Gao and Bhattacharyya, 2008; Cook et al., 2012; Liu et al., 2012; Lu et al., 2017; Ma et al., 2017), the functions of many soybean genes remain unknown. As the demand for soybean is increasing, how to improve soybean yield and edible quality is an urgent challenge confronted by soybean breeders.

### Rationality and Advantage of Our Strategy for Studies of Soybean Functional Genomics

Currently, when we mine favorable genes in soybean through the method of reverse genetics, we usually select dozens of candidate genes from transcriptome data or known homologous genes, and then investigate their functions one by one by use of *Arabidopsis* as the model plant. Subsequently, we confirm their functions in soybean. This process requires large amounts of time and labor, and the positive efficiency is relatively low, which severely influences the mining of favorable genes. On the other hand, soybean is an ancient tetraploid plant, and most of the soybean genes have duplicated copies, meaning that loss-of-function mutations in soybean genes usually have no apparent phenotypes. In addition, the successes of QTL cloning in soybean have been few to date, largely due to the complexity of the soybean genome (Kato et al., 2014; Lee et al., 2015). Due to the above, elucidation of the functions of soybean genes and, further, the mining of favorable genes have been much slower than those in other crops. Developing a large-scale and efficient strategy for studies of soybean functional genomics is an urgent need for soybean researchers.

*Arabidopsis* has diverse genetic resources and a large number of mutants created using various mutagenesis strategies^2^. Moreover, *Arabidopsis* has a small genome, short life cycle, and easy and effective transformation, making *Arabidopsis* a useful model plant (Koornneef and Meinke, 2010; Liepman et al., 2010). To date, many gene functions in *Arabidopsis* have been dissected and provided important references for other plant species^3^. Both soybean and *Arabidopsis* are dicotyledonous plants and share some similarities. These similarities provide a substantial potential for the use of *Arabidopsis* to carry out studies of soybean functional genomics. By using *Arabidopsis* as a model plant, the functions of many soybean genes have already been successfully elucidated (Li et al., 2016; Takeshima et al., 2016; Chu et al., 2017). For example, *GmNAC20* and *GmNAC11* are two NAC transcription factors in soybean. When *GmNAC20* was overexpressed in *Arabidopsis*, it greatly enhanced salt and freezing tolerance, whereas overexpression of *GmNAC11* improved only salt tolerance in transgenic *Arabidopsis*. The roles of these two NAC family transcription factors were also confirmed in soybean (Hao et al., 2011).

To mine gene functions at a larger scale, we constructed a full-length soybean cDNA library with a modified overexpression vector, *pJL12*, which contains a 35S promoter (**Figure 1A**). We assessed the resulting library and found that it had high quality (**Figure 2**). After transformation into *Arabidopsis*, we first obtained ∼2000 transgenic lines (**Table 2**), among which 5% of transgenic plants showed abnormal developmental phenotypes, whereas 1% of them showed potentially beneficial traits (**Figure 3** and **Table 2**). Sequence analyses showed that all overexpressed soybean genes were different rather than redundant, suggesting the effectiveness of our strategy. We noticed that some soybean genes could be missed, including genes that exhibit phenotypic changes only in the loss-of-function condition and genes that have different functions in soybean from those in *Arabidopsis*. Moreover, some phenotypes exhibited by the transgenic *Arabidopsis* lines might be attributed to the insertion of vectors in the *Arabidopsis* genome rather than the overexpression of soybean genes. In addition, ectopic overexpression of a gene might not reflect its intrinsic function, which could be solved by studying the corresponding loss-of-function mutant or using the native promoter. Despite these imperfections, our results prove that this strategy is reasonable and advantageous for use in investigating the functions of soybean genes and further mining favorable soybean genes for future molecular improvements.

### Dissection of Four Potentially Favorable Soybean Genes

In this study, we transformed the soybean full-length cDNA overexpression library into *Arabidopsis*. Many T_1_ generation positive seedlings exhibited various phenotypes (**Table 2**). The occurrences of phenotypes in these transgenic lines might be attributed to the overexpression of the soybean genes or to the destruction of *Arabidopsis* gene expression by T-DNA insertion in the *Arabidopsis* genome.

To confirm that the phenotypes occurring in the T_1_ positive seedlings were caused by cDNA ectopic overexpression, we randomly selected 4 lines for confirmation. Genetic retransformation proved that the overexpressed soybean cDNAs were responsible for each of the observed phenotypes. A13 encodes an *Arabidopsis* SGR1 homolog protein. In *Arabidopsis*, SGR1 interacts with Chl catabolic enzymes (CCE) to regulate chlorophyll metabolism, leading to a pale and yellowing leaf pattern during leaf senescence (Balazadeh, 2014; Sakuraba et al., 2014a,b, 2015; Bell et al., 2015). Furthermore, *SGR1* regulates chlorophyll degradation in other plant species, such as rice (Park et al., 2007), pea (*Pisum sativum*) (Sato et al., 2007), tomato (*Solanum lycopersicum*), bell pepper (*Capsicum annuum*) (Barry et al., 2008), tall fescue (*Festuca arundinacea*) (Wei et al., 2011) and *Medicago truncatula* (Zhou et al., 2011). In this study, it also caused a yellowish appearance, indicating a conserved role of *SGR1*. B12 encodes an ANDROGEN INDUCED INHIBITOR OF PROLIFERATION (AS3) / PDS5-RELATED protein, which may inhibit cell proliferation, consistent with the dwarfing and highly branched phenotype of B12 (Yuan et al., 1993; Szelei et al., 1997). C15 encodes an ATP-dependent RNA helicase. RNA helicase plays diverse roles in plant growth and development (Linder and Owttrim, 2009), which may be a plausible reason for the small stature and early-flowering phenotype of C15. D70 encodes an aldehyde dehydrogenase (Brocker et al., 2013), which may affect the metabolism of energy in seeds. All of these genes are worthy of further study to elucidate their detailed functional mechanisms.

## Conclusion

In this study, we used *Arabidopsis* as a recipient and developed a high-throughput strategy to investigate the functions of soybean genes by overexpressing a full-length soybean cDNA library in *Arabidopsis*. Many transgenic *Arabidopsis* lines exhibited abnormal phenotypes, among which some were found to contain potentially favorable genes for the molecular improvement of soybean. Although the functions of these genes must be further confirmed in soybean, we believe that this strategy may provide valuable sources of soybean genetic improvement.

## Author Contributions

XL performed the majority of the experimental work. LH helped to construct the cDNA library. JL conducted part of the transformation work. YC constructed the overexpression vector. QY, LW, XS, and XZ gave valuable advice. XL and YJ wrote and revised the paper. YJ conceived the project and supervised the work.

## Conflict of Interest Statement

The authors declare that the research was conducted in the absence of any commercial or financial relationships that could be construed as a potential conflict of interest.

## Footnotes

1 http://www.soybase.org/

2 http://signal.salk.edu/cgi-bin/tdnaexpress

3 http://www.arabidopsis.org/

## References

[B1] Balazadeh S. (2014). Stay-green not always stays green. 7 1264–1266. 10.1093/mp/ssu076 24996917

[B2] Barry C. S. Mcquinn R. P. Chung M. Y. Besuden A. Giovannoni J. J. (2008). Amino acid substitutions in homologs of the STAY-GREEN protein are responsible for the green-flesh and chlorophyll retainer mutations of tomato and pepper. 147 179–187. 10.1104/pp.108.118430 18359841 PMC2330295

[B3] Bell A. Moreau C. Chinoy C. Spanner R. Dalmais M. Le Signor C. (2015). SGRL can regulate chlorophyll metabolism and contributes to normal plant growth and development in *Pisum sativum* L. 89 539–558. 10.1007/s11103-015-0372-4 26346777 PMC4659853

[B4] Brocker C. Vasiliou M. Carpenter S. Carpenter C. Zhang Y. Wang X. (2013). Aldehyde dehydrogenase (ALDH) superfamily in plants: gene nomenclature and comparative genomics. 237 189–210. 10.1007/s00425-012-1749-0 23007552 PMC3536936

[B5] Chi Y. Huang F. Liu H. Yang S. Yu D. (2011). An APETALA1-like gene of soybean regulates flowering time and specifies floral organs. 168 2251–2259. 10.1016/j.jplph.2011.08.007 21963279

[B6] Chu S. Wang J. Zhu Y. Liu S. Zhou X. Zhang H. (2017). An R2R3-type MYB transcription factor, GmMYB29, regulates isoflavone biosynthesis in soybean. 13:e1006770. 10.1371/journal.pgen.1006770 28489859 PMC5443545

[B7] Clough S. J. Bent A. F. (1998). Floral dip: a simplified method for Agrobacterium-mediated transformation of *Arabidopsis thaliana*. 16 735–743. 10.1046/j.1365-313x.1998.00343.x 10069079

[B8] Collins N. C. Thordal-Christensen H. Lipka V. Bau S. Kombrink E. Qiu J. L. (2003). SNARE-protein-mediated disease resistance at the plant cell wall. 425 973–977. 10.1038/nature02076 14586469

[B9] Cook D. E. Lee T. G. Guo X. Melito S. Wang K. Bayless A. M. (2012). Copy number variation of multiple genes at Rhg1 mediates nematode resistance in soybean. 338 1206–1209. 10.1126/science.1228746 23065905

[B10] Gao H. Bhattacharyya M. K. (2008). The soybean-Phytophthora resistance locus Rps1-k encompasses coiled coil-nucleotide binding-leucine rich repeat-like genes and repetitive sequences. 8:29. 10.1186/1471-2229-8-29 18366691 PMC2330051

[B11] Gao H. Narayanan N. N. Ellison L. Bhattacharyya M. K. (2005). Two classes of highly similar coiled coil-nucleotide binding-leucine rich repeat genes isolated from the Rps1-k locus encode *Phytophthora* resistance in soybean. 18 1035–1045. 10.1094/MPMI-18-1035 16255242

[B12] Guo W. Zuo Z. Cheng X. Sun J. Li H. Li L. (2014). The chloride channel family gene CLCd negatively regulates pathogen-associated molecular pattern (PAMP)-triggered immunity in *Arabidopsis*. 65 1205–1215. 10.1093/jxb/ert484 24449384 PMC3935575

[B13] Hao Y. J. Wei W. Song Q. X. Chen H. W. Zhang Y. Q. Wang F. (2011). Soybean NAC transcription factors promote abiotic stress tolerance and lateral root formation in transgenic plants. 68 302–313. 10.1111/j.1365-313X.2011.04687.x 21707801

[B14] Jiao Y. Wang Y. Xue D. Wang J. Yan M. Liu G. (2010). Regulation of OsSPL14 by OsmiR156 defines ideal plant architecture in rice. 42 541–544. 10.1038/ng.591 20495565

[B15] Kato S. Sayama T. Fujii K. Yumoto S. Kono Y. Hwang T. Y. (2014). A major and stable QTL associated with seed weight in soybean across multiple environments and genetic backgrounds. 127 1365–1374. 10.1007/s00122-014-2304-0 24718925

[B16] Koornneef M. Meinke D. (2010). The development of Arabidopsis as a model plant. 61 909–921. 10.1111/j.1365-313X.2009.04086.x 20409266

[B17] Korir P. C. Zhang J. Wu K. Zhao T. Gai J. (2013). Association mapping combined with linkage analysis for aluminum tolerance among soybean cultivars released in Yellow and Changjiang River Valleys in China. 126 1659–1675. 10.1007/s00122-013-2082-0 23515677

[B18] Lam H. M. Xu X. Liu X. Chen W. Yang G. Wong F. L. (2010). Resequencing of 31 wild and cultivated soybean genomes identifies patterns of genetic diversity and selection. 42 1053–1059. 10.1038/ng.715 21076406

[B19] Lanubile A. Muppirala U. K. Severin A. J. Marocco A. Munkvold G. P. (2015). Transcriptome profiling of soybean (*Glycine max*) roots challenged with pathogenic and non-pathogenic isolates of *Fusarium oxysporum*. 16:1089. 10.1186/s12864-015-2318-2 26689712 PMC4687377

[B20] Lee G. J. Carter TE Jr Villagarcia M. R. Li Z. Zhou X. Gibbs M. O. (2004). A major QTL conditioning salt tolerance in S-100 soybean and descendent cultivars. 109 1610–1619. 10.1007/s00122-004-1783-9 15365627

[B21] Lee J. S. Yoo M. H. Jung J. K. Bilyeu K. D. Lee J. D. Kang S. (2015). Detection of novel QTLs for foxglove aphid resistance in soybean. 128 1481–1488. 10.1007/s00122-015-2519-8 25904004

[B22] Li Q. Fang C. Duan Z. Liu Y. Qin H. Zhang J. (2016). Functional conservation and divergence of GmCHLI genes in polyploid soybean. 88 584–596. 10.1111/tpj.13282 27459730

[B23] Li Q. T. Lu X. Song Q. X. Chen H. W. Wei W. Tao J. J. (2017). Selection for a zinc-finger protein contributes to seed oil increase during soybean domestication. 173 2208–2224. 10.1104/pp.16.01610 28184009 PMC5373050

[B24] Li Z. C. An L. H. Fu Q. Liu Y. Zhang L. Chen H. (2012). Construction and characterization of a normalized cDNA library from the river snail Bellamya aeruginosa after exposure to copper. 21 260–267. 10.1007/s10646-011-0786-y 21915736

[B25] Lichtenthaler H. K. (1987). Chlorophylls and carotenoids: pigments of photosynthetic biomembranes. 148 350–382. 10.1016/0076-6879(87)48036-1

[B26] Liepman A. H. Wightman R. Geshi N. Turner S. R. Scheller H. V. (2010). Arabidopsis - a powerful model system for plant cell wall research. 61 1107–1121. 10.1111/j.1365-313X.2010.04161.x 20409281

[B27] Linder P. Owttrim G. W. (2009). Plant RNA helicases: linking aberrant and silencing RNA. 14 344–352. 10.1016/j.tplants.2009.03.007 19446493

[B28] Liu B. Kanazawa A. Matsumura H. Takahashi R. Harada K. Abe J. (2008). Genetic redundancy in soybean photoresponses associated with duplication of the phytochrome A gene. 180 995–1007. 10.1534/genetics.108.092742 18780733 PMC2567397

[B29] Liu S. Kandoth P. K. Warren S. D. Yeckel G. Heinz R. Alden J. (2012). A soybean cyst nematode resistance gene points to a new mechanism of plant resistance to pathogens. 492 256–260. 10.1038/nature11651 23235880

[B30] Liu Y. F. Li Q. T. Lu X. Song Q. X. Lam S. M. Zhang W. K. (2014). Soybean GmMYB73 promotes lipid accumulation in transgenic plants. 14:73. 10.1186/1471-2229-14-73 24655684 PMC3998039

[B31] Lu S. Zhao X. Hu Y. Liu S. Nan H. Li X. (2017). Natural variation at the soybean J locus improves adaptation to the tropics and enhances yield. 49 773–779. 10.1038/ng.3819 28319089

[B32] Ma Z. Zhu L. Song T. Wang Y. Zhang Q. Xia Y. (2017). A paralogous decoy protects *Phytophthora sojae* apoplastic effector PsXEG1 from a host inhibitor. 355 710–714. 10.1126/science.aai7919 28082413

[B33] Martinez-Trujillo M. Limones-Briones V. Cabrera-Ponce J. L. Herrera-Estrella L. (2004). Improving transformation efficiency of *Arabidopsis thaliana* by modifying the floral dip method. 22 63–70. 10.1007/BF02773350

[B34] Pan W. J. Tao J. J. Cheng T. Shen M. Ma J. B. Zhang W. K. (2017). Soybean NIMA-related kinase1 promotes plant growth and improves salt and cold tolerance. 58 1268–1278. 10.1093/pcp/pcx060 28444301

[B35] Park S. Y. Yu J. W. Park J. S. Li J. Yoo S. C. Lee N. Y. (2007). The senescence-induced stay green protein regulates chlorophyll degradation. 19 1649–1664. 10.1105/tpc.106.044891 17513504 PMC1913741

[B36] Patanjali S. R. Parimoo S. Weissman S. M. (1991). Construction of a uniform-abundance (normalized) cDNA library. 88 1943–1947. 10.1073/pnas.88.5.1943 1705712 PMC51142

[B37] Qiu J. L. Fiil B. K. Petersen K. Nielsen H. B. Botanga C. J. Thorgrimsen S. (2008a). *Arabidopsis* MAP kinase 4 regulates gene expression through transcription factor release in the nucleus. 27 2214–2221. 10.1038/emboj.2008.147 18650934 PMC2519101

[B38] Qiu J. L. Jilk R. Marks M. D. Szymanski D. B. (2002). The Arabidopsis SPIKE1 gene is required for normal cell shape control and tissue development. 14 101–118. 10.1105/tpc.010346 11826302 PMC150554

[B39] Qiu J. L. Zhou L. Yun B. W. Nielsen H. B. Fiil B. K. Petersen K. (2008b). Arabidopsis mitogen-activated protein kinase kinases MKK1 and MKK2 have overlapping functions in defense signaling mediated by MEKK1, MPK4, and MKS1. 148 212–222. 10.1104/pp.108.120006 18599650 PMC2528087

[B40] Sakuraba Y. Kim D. Kim Y. S. Hortensteiner S. Paek N. C. (2014a). Arabidopsis STAYGREEN-LIKE (SGRL) promotes abiotic stress-induced leaf yellowing during vegetative growth. 588 3830–3837. 10.1016/j.febslet.2014.09.018 25261252

[B41] Sakuraba Y. Park S. Y. Kim Y. S. Wang S. H. Yoo S. C. Hortensteiner S. (2014b). *Arabidopsis* STAY-GREEN2 is a negative regulator of chlorophyll degradation during leaf senescence. 7 1288–1302. 10.1093/mp/ssu045 24719469

[B42] Sakuraba Y. Park S. Y. Paek N. C. (2015). The divergent roles of STAYGREEN (SGR) homologs in chlorophyll degradation. 38 390–395. 10.14348/molcells.2015.0039 25913011 PMC4443279

[B43] Sato Y. Morita R. Nishimura M. Yamaguchi H. Kusaba M. (2007). Mendel’s green cotyledon gene encodes a positive regulator of the chlorophyll-degrading pathway. 104 14169–14174. 10.1073/pnas.0705521104 17709752 PMC1955798

[B44] Schmutz J. Cannon S. B. Schlueter J. Ma J. Mitros T. Nelson W. (2010). Genome sequence of the palaeopolyploid soybean. 463 178–183. 10.1038/nature08670 20075913

[B45] Shimoda Y. Ito H. Tanaka A. (2016). Arabidopsis STAY-GREEN, mendel’s green cotyledon gene, encodes magnesium-dechelatase. 28 2147–2160. 10.1105/tpc.16.00428 27604697 PMC5059807

[B46] Si L. Chen J. Huang X. Gong H. Luo J. Hou Q. (2016). OsSPL13 controls grain size in cultivated rice. 48 447–456. 10.1038/ng.3518 26950093

[B47] Smith-Hammond C. L. Swatek K. N. Johnston M. L. Thelen J. J. Miernyk J. A. (2014). Initial description of the developing soybean seed protein Lys-N(epsilon)-acetylome. 96 56–66. 10.1016/j.jprot.2013.10.038 24211405

[B48] Soares M. B. Bonaldo M. F. Jelene P. Su L. Lawton L. Efstratiadis A. (1994). Construction and characterization of a normalized cDNA library. 91 9228–9232. 10.1073/pnas.91.20.9228 PMC44785 7937745

[B49] Sobhanian H. Razavizadeh R. Nanjo Y. Ehsanpour A. A. Jazii F. R. Motamed N. (2010). Proteome analysis of soybean leaves, hypocotyls and roots under salt stress. 8:19. 10.1186/1477-5956-8-19 20350314 PMC2859372

[B50] Song X. J. Huang W. Shi M. Zhu M. Z. Lin H. X. (2007). A QTL for rice grain width and weight encodes a previously unknown RING-type E3 ubiquitin ligase. 39 623–630. 10.1038/ng2014 17417637

[B51] Szelei J. Jimenez J. Soto A. M. Luizzi M. F. Sonnenschein C. (1997). Androgen-induced inhibition of proliferation in human breast cancer MCF7 cells transfected with androgen receptor. 138 1406–1412. 10.1210/endo.138.4.5047 9075695

[B52] Takeshima R. Hayashi T. Zhu J. Zhao C. Xu M. Yamaguchi N. (2016). A soybean quantitative trait locus that promotes flowering under long days is identified as FT5a, a FLOWERING LOCUS T ortholog. 67 5247–5258. 10.1093/jxb/erw283 27422993 PMC5014162

[B53] Wang F. Chen H. W. Li Q. T. Wei W. Li W. Zhang W. K. (2015). GmWRKY27 interacts with GmMYB174 to reduce expression of GmNAC29 for stress tolerance in soybean plants. 83 224–236. 10.1111/tpj.12879 25990284

[B54] Wang S. Li S. Liu Q. Wu K. Zhang J. Wang Y. (2015). The OsSPL16-GW7 regulatory module determines grain shape and simultaneously improves rice yield and grain quality. 47 949–954. 10.1038/ng.3352 26147620

[B55] Wang Y. Xiong G. Hu J. Jiang L. Yu H. Xu J. (2015). Copy number variation at the GL7 locus contributes to grain size diversity in rice. 47 944–948. 10.1038/ng.3346 26147619

[B56] Wei Q. Guo Y. Kuai B. (2011). Isolation and characterization of a chlorophyll degradation regulatory gene from tall fescue. 30 1201–1207. 10.1007/s00299-011-1028-8 21327390

[B57] Wei W. Li Q. T. Chu Y. N. Reiter R. J. Yu X. M. Zhu D. H. (2015). Melatonin enhances plant growth and abiotic stress tolerance in soybean plants. 66 695–707. 10.1093/jxb/eru392 25297548 PMC4321538

[B58] Xia Z. Zhai H. Lu S. Wu H. Zhang Y. (2013). Recent achievement in gene cloning and functional genomics in soybean. 2013:281367. 10.1155/2013/281367 24311973 PMC3842071

[B59] Yu X. Yuan F. Fu X. Zhu D. (2016). Profiling and relationship of water-soluble sugar and protein compositions in soybean seeds. 196 776–782. 10.1016/j.foodchem.2015.09.092 26593554

[B60] Yuan S. Trachtenberg J. Mills G. B. Brown T. J. Xu F. Keating A. (1993). Androgen-induced inhibition of cell proliferation in an androgen-insensitive prostate cancer cell line (PC-3) transfected with a human androgen receptor complementary DNA. 53 1304–1311. 8443809

[B61] Zhang Z. X. Zhang F. D. Tang W. H. Pi Y. J. Zheng Y. L. (2005). [Construction and characterization of normalized cDNA library of maize inbred Mo17 from multiple tissues and developmental stages]. 39 198–206. 10.1007/s11008-005-0026-8 15856942

[B62] Zhou C. Han L. Pislariu C. Nakashima J. Fu C. Jiang Q. (2011). From model to crop: functional analysis of a STAY-GREEN gene in the model legume *Medicago truncatula* and effective use of the gene for alfalfa improvement. 157 1483–1496. 10.1104/pp.111.185140 21957014 PMC3252161

[B63] Zou L. P. Sun X. H. Zhang Z. G. Liu P. Wu J. X. Tian C. J. (2011). Leaf rolling controlled by the homeodomain leucine zipper class IV gene Roc5 in rice. 156 1589–1602. 10.1104/pp.111.176016 21596949 PMC3135938

