# Supplementary material for: Large-Scale Investigation of Soybean Gene Functions by Overexpressing a Full-Length Soybean cDNA Library in Arabidopsis
Source: Front Plant Sci. 2018 May 9;9:631. doi: 10.3389/fpls.2018.00631 (PMC5954216; doi:10.3389/fpls.2018.00631)
Supplement: Supplementary file 1 [file Presentation_1.PDF]

Supplementary file 1 Primer sequences used in phenotypic validation and plasmid construction.

primers	sequence
A13-f	atctgatcaagagacaggatcc ATGGCATGTCATTGTGCTTCT
A13-r	cgctctagaactagtggatccTCAAAGAAGAAAAGCAAAAAG
B12-f	atctgatcaagagacaggatcc ATGGCAATGGCGTCTACCGA
B12-r	cgctctagaactagtggatccTCAAACATCAGAGTTCT
C15-f	atctgatcaagagacaggatcc ATGTCGTATGTGCCTCCGCA
C15-r	cgctctagaactagtggatccTTAATCATCCCAGGCACT
C21-f	atctgatcaagagacaggatcc ATGGCAAGTGGCTCGGAC
C21-r	cgctctagaactagtggatccTTATATTGCGAGAACCAAA
D70-f	atctgatcaagagacaggatcc ATGTTCTTGTCTTTGCATATAG
D70-r	CgctctagaactagtggatccTCAAAGCCAAGGAGAATTATG
pJL12-f	GACGCACAATCCCACTATC
pJL12-r	GCCAATATATCCTGTCAAACACTG
