# Supplementary material for: Large-Scale Investigation of Soybean Gene Functions by Overexpressing a Full-Length Soybean cDNA Library in Arabidopsis
Source: Front Plant Sci. 2018 May 9;9:631. doi: 10.3389/fpls.2018.00631 (PMC5954216; doi:10.3389/fpls.2018.00631)
Supplement: Supplementary file 2 [file Presentation_2.PDF]

Supplementary file 2 Artificial synthesized sequence for modification of vector pJL2.

ggatccggccattacggccaagcttgatatcggccgcctcggccgcggccgccac
cgcggtggagctcgaatttccccgatcgttcaaacatttggcaataaagtttcttaaga
ttgaatcctgttgccggtcttgcgatgattatcatataatttctgttgaattacgttaa
gcatgtaataattaacatgtaatgcatgacgttatttatgagatgggtttttatgatta
gagtcccgcaattatacatttaatacgcgatagaaaacaaaatatagcgcgcaaactag
gataaattatcgcgcgcggtgtcatctatgttactagatcgggaattagctcagattgt
cgtttcccgccttcagtttaaactatcagtgtttgacaggatatattggcgggtaaacc
taagagaaaagagcgtttattagaataatcggatatttaaaagggcgtgaaaaggttta
tccgttcgtccatttgtatgtgcatgccaaccacaggagatctcagtaaagcgctggct
gctgaaccccagccggaactgaccccacaaggccctagcgtttgcaatgcaccaggtc
atcattgacccaggcgtgttccaccaggccgctgcctcgcaactcttcgcaggcttcgc
cgacctgctcgcgccacttcttcacgcgggtggaatccgatccgcacatgaggcggaag
gtttccagcttgagcgggtacggctcccggtgcgagctgaaatagtcgaacatccgtcg
ggccgtcggcgacagcttgcggtacttctcccatatgaatttcgtgtagtggtcgccag
caaacagcacgacgatttcctcgtcgatcaggacctggcaacgggacgttttcttgcca
cggtccaggacgcggaagcggtgcagcagcgacaccgattccaggtgcccaacgcggtc
ggacgtgaagcccatcgccgtcgcctgtaggcgcgacaggcattcctcggccttcgtgt
aataccggccattgatcgaccagcccaggtcctggcaaagctcgtagaacgtgaaggtg
atcggctcgccgataggggtgcgcttcgcgtactccaacacctgctgccacaccagttc
gtcatcgtcggcccgcagctcgacgccggtgtaggtgatcttcacgtccttgttgacgt
ggaaaatgaccttgttttgcagcgcctcgcgcgggattttcttgttgcgcgtggtgaac
agggcagagcgggccgtgtcgtttggcatcgctcgcatcgtgtccggccacggcgcaat
atcgaacaaggaaagctgcatttccttgatctgctgcttcgtgtgtttcagcaacgcgg
cctgctttgcctcgctgacctgttttgccaggtcctcgccggcg

The artificial synthesized sequence was digested by *BamHI* and *Sse232I* and linked to vector *pJL12* to replace sequence between 2563-3927 positions. The yellow backgrounds indicate *BamHI* (upper) and *Sse232I* (lower) recognition site respectively. The green backgrounds indicate substitute nucleotide, and the violet backgrounds indicate the additional added *sfiI* site. The underlined letters indicate initial *sfiI* recognition site in *pJL12* vector.
