# Supplementary material for: Large-Scale Investigation of Soybean Gene Functions by Overexpressing a Full-Length Soybean cDNA Library in Arabidopsis
Source: Front Plant Sci. 2018 May 9;9:631. doi: 10.3389/fpls.2018.00631 (PMC5954216; doi:10.3389/fpls.2018.00631)
Supplement: Supplementary file 3 [file Presentation_3.PDF]

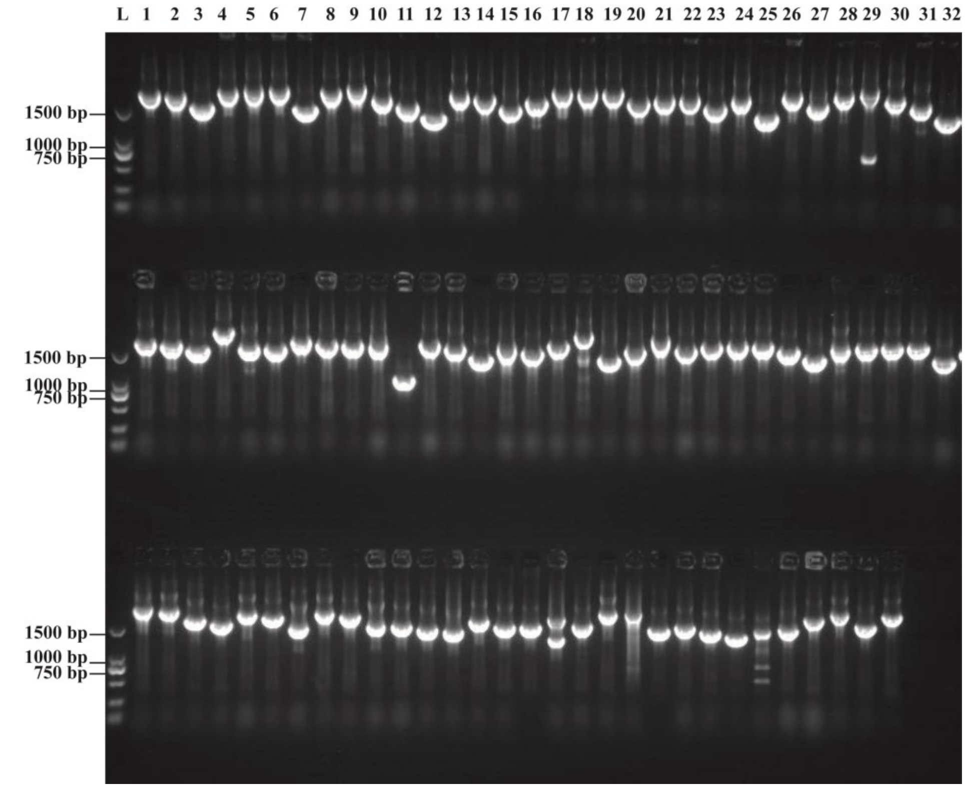

Supplementary file 3 Agarose gel electrophoresis of 96 selected colonies from the constructed soybean full-length cDNA overexpression library. Primers PJL12-f and PJL12-r were used to amplify cDNAs inserted into modified vector *PJL12*. L: molecular ladder.
