# Supplementary material for: Large-Scale Investigation of Soybean Gene Functions by Overexpressing a Full-Length Soybean cDNA Library in Arabidopsis
Source: Front Plant Sci. 2018 May 9;9:631. doi: 10.3389/fpls.2018.00631 (PMC5954216; doi:10.3389/fpls.2018.00631)
Supplement: Supplementary file 4 [file Presentation_4.PDF]

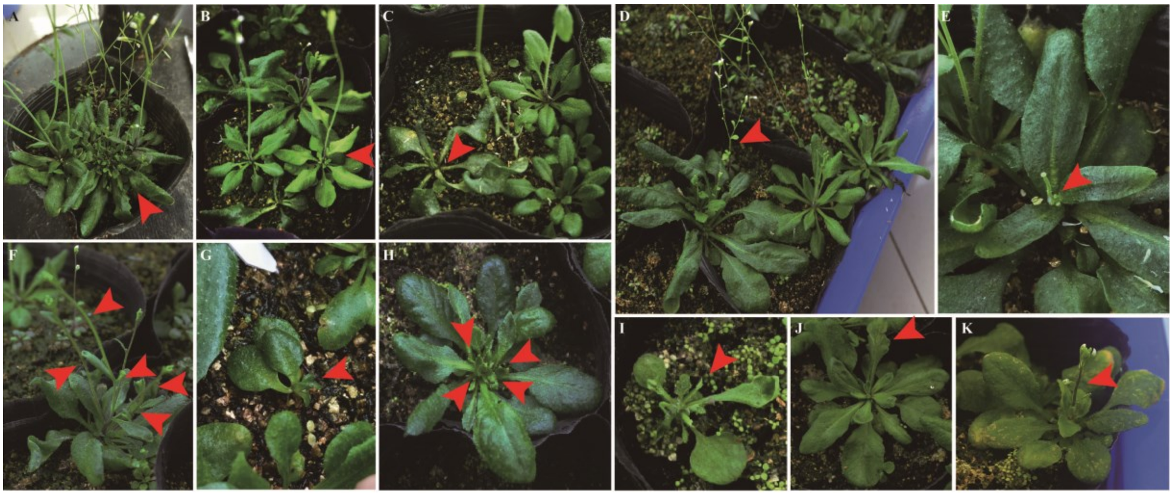

Supplementary file 4 Positive T1 transgenic Arabidopsis exhibited diverse developmental defects with high frequency. A. Late flowering. B. Yellowish plant. C. Loss of SAM activity. D. Early senescence. E. No seed stalk but only one silique. F. More secondary branches. G. Abnormal asymmetrical growth. H. More primary branches. I. Irregular clavate leaf shape. J. Serrated leaf shape. K. Smooth stem. Red arrows indicate appeared abnormal phenotypes.
