# Supplementary material for: Large-Scale Investigation of Soybean Gene Functions by Overexpressing a Full-Length Soybean cDNA Library in Arabidopsis
Source: Front Plant Sci. 2018 May 9;9:631. doi: 10.3389/fpls.2018.00631 (PMC5954216; doi:10.3389/fpls.2018.00631)
Supplement: Supplementary file 5 [file Presentation_5.PDF]

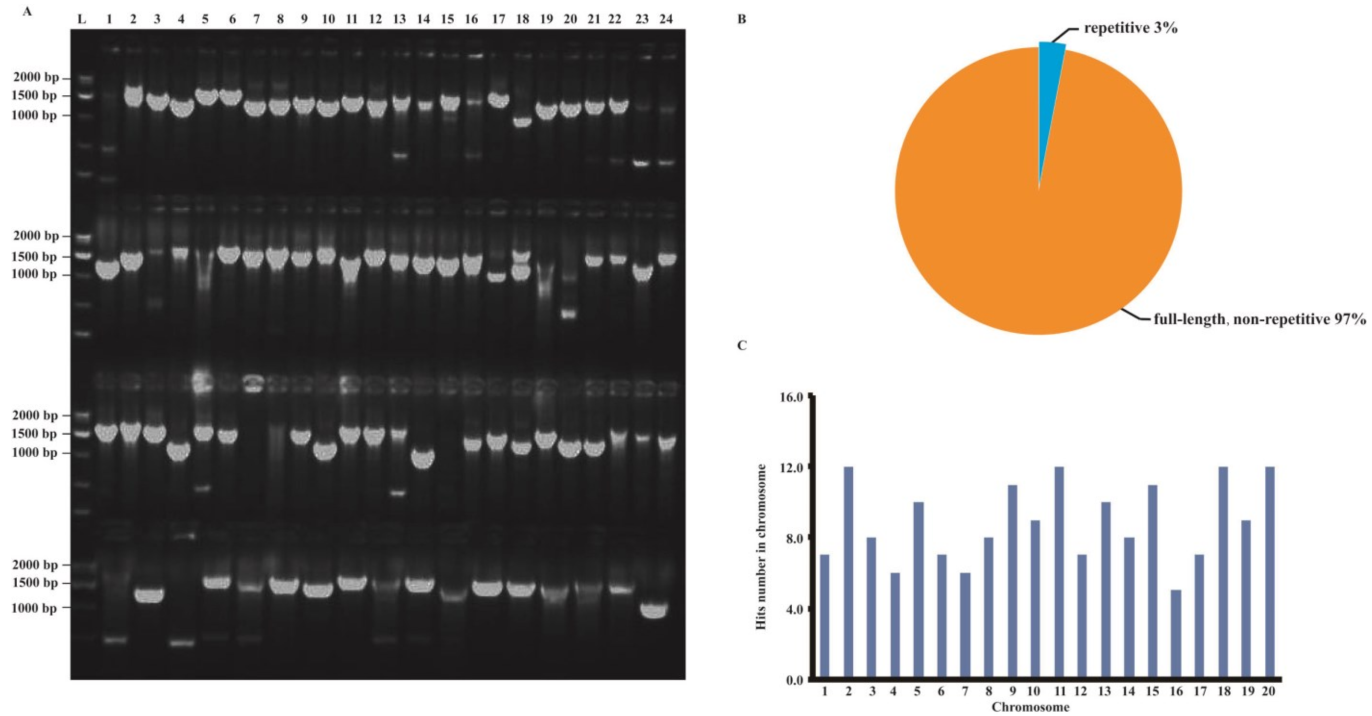

Supplementary file 5 Examination of cDNA inserts in 96 selected T_1 positive seedlings. A. PCR amplification and agarose gel electrophoresis of 96 selected positive T_1 seedlings using primers PJL12-f and PJL12-r. B. Full length analysis and normalization verification on sequencing data. C. Distribution analysis of sequencing data on soybean 20 chromosomes.
